# Travel risk behaviors as a determinants of receiving pre-travel health consultation and prevention

**DOI:** 10.1186/s40794-015-0003-8

**Published:** 2015-07-29

**Authors:** Ibrahim Shady, Mohammed Gaafer, Lamiaa Bassiony

**Affiliations:** 1 Faculty of Medicine, Mansoura University, 35516 Mansoura, Egypt; 2 Faculty of Medicine, Menoufia University, Menoufia, Egypt

**Keywords:** Travel Health, International Health, International Health Regulation, Malaria prophylaxis, Yellow fever, Vaccination

## Abstract

**Background:**

An estimated 30-60 % of travelers experience an illness while traveling. The incidence of travel-related illness can be reduced by preventive measures such as those provided by the Traveler Health Clinic (THC) in Kuwait.

**Methods:**

The present study is an analytical comparative study between groups of travelers visiting the THC during the study period (May 2009 – December 2010) and an age- and gender-matched control group of non-visitors (800 people). Both groups completed a modified pre-departure questionnaire.

**Results:**

Bivariate analysis revealed that Kuwaitis (68.2 %), those traveling for work (25.3 %) or leisure (59.5 %), those living in camps (20.4 %) or hotels (64.0 %), and those with knowledge of the THC from the media (28.1 %) or other sources (57.3 %), were more likely to be associated with a high frequency of visits to the THC (*p* < 0.001). Additionally, travelers heading to Africa (47 %) and South America (10 %) visited the THC more than did others (*P* < 0.05). Multivariate analysis revealed that nationality, followed by purpose of travel, duration of stay, and choice of travel destination are independent predictors of receiving pre-travel consultation from the THC.

**Conclusion:**

Nationality, purpose of travel, length of stay, and travel destination are predictors for receiving a pre-travel consultation from the THC.

**Electronic supplementary material:**

The online version of this article (doi:10.1186/s40794-015-0003-8) contains supplementary material, which is available to authorized users.

## Background

International travel has recently increased in magnitude, speed, and geographical reach, with 940 million arrivals reported in 2010. Every year, as many as 50 million people from the industrialized world cross international borders to tropical or subtropical destinations. These travelers encounter different cultures, social habits, economic standards, and, importantly, a different microbiological environment [1, 2].

The State of Kuwait is a country with a total population of 3.6 million, including approximately 1.2 million Kuwaiti citizens and 2.4 million non-Kuwaitis [3].

An estimated 700 travelers (2–3 visits/10,000 population) visit the Traveler Health Clinic every year. The Traveler Health Clinic (THC) is the only clinic in Kuwait that is accessible by a variety of transportation methods because of its location in the middle of Kuwait City.

The services provided at the THC include immunization, chemoprophylaxis for malaria, and health advice/information for the prevalent diseases/health hazards in the destination country. These services are provided to travelers free of charge, except for yellow fever vaccination [4].

The volume of travelers from Kuwait varies throughout the year and has two peaks: during the summer months and during the midterm vacations of schools and universities [4].

Despite public health efforts designed to make travelers aware of the risks related to international travel, the number of travelers who consult travel clinics remains relatively low. Some studies of travel risk behaviors have reported that between 4 % and 52 % of travelers sought pre-travel advice at travel clinics [5–13].

Although some studies focus on the factors underlying seeking consultation prior to travel, associations between travel clinic consultations and other variables have been brought to the forefront. Time constraints and limited geographical or financial accessibility may constitute barriers to obtaining pre-travel information. Socio-demographic characteristics (e.g., age, sex, education level, and socioeconomic status) do not seem to be significantly associated with seeking pre-travel consultation and prevention, whereas travel attributes (e.g., destination, reason for travel, and trip duration) and traveler characteristics seem to play more important roles [10–15].

To the authors’ best knowledge, no studies have been performed in the Gulf region that address traveler health-seeking behaviors and their determinants. The objectives of this study are to identify the determinants of receiving pre-travel health consultation and the contents of this care.

## Patients and Methods

This study is an analytical, comparative study involving demographic and travel data for travelers visiting the THC (visitor group) and a matched control group (non-visitor group).

The study period was from May 1, 2009 to December 31, 2010.

### Participants from the Kuwait Travel Health Clinic (Visitor Group)

The study cohort comprised travelers visiting the Traveler Health Clinic (THC) in Kuwait.

- The researchers and assistants (physicians with doctorate degrees in public health, nurses, and health inspectors) interviewed the travelers who visited the clinic and asked them to fill out the pre-departure health questionnaire [16] (Additional file 1).
- Informed verbal consent was obtained from the participants of the study before they began. They had been informed about the importance of the study and the steps they will go through during the study and all accepted to participate in it. Children were consented through their parents, brother/sisters or any accompanying relatives.
- Each traveler’s interview lasted 15–30 min.
- The validity of the questionnaires was tested, and missing items were completed by the traveler himself or herself after receiving preventive measures from the clinic.
- Preventive measures administered to the travelers in accordance with the World Health Organization (WHO) rules and regulations and International Health Regulations (IHR 2005) [16].

### Participants from the airport (Non-visitors group)

Age- and gender-matched controls (800 travelers) were chosen from a group of travelers in the airport who had finished boarding procedures. The required numbers of controls were calculated according to the weekly proportions of the ages and genders of the visitor group.

The THC is open Sunday through Thursday, and Friday and Saturday are considered the weekend. At the end of the workweek (Thursday), we received the weekly number of participants from the visitors’ group. Then, we chose an equivalent number of participants from the airport during the weekend using systematic random sampling (every 5^th^ traveler) at a 1:1 case/control ratio. If one selectee had visited the THC, the next passenger was chosen.

- Exclusion criteria included non-resident visitors to Kuwait and permanent travelers to their home country.
- We designed two versions of the questionnaire (Arabic and English).
- Travelers under 6 years of age were helped by their accompanying adults. Those who could not understand or fill out the questionnaire were helped by the clinic's staff members.
- The questionnaire included socio-demographic data (gender, age, nationality, and occupation), data from the trip (destination, purpose of travel, date of travel, length of stay, locations to be visited, activities planned during the visit, accommodations, history of past travel), and source of knowledge of the THC.
- Occupations were classified into four categories: Unemployed, Domestic Worker, White Collar Occupation (refers to a person who performs professional, managerial, or administrative work in an office or cubicle), and Blue Collar Occupation (manual labor).
- Prior to the study, the required organizational was obtained from the traveler health board and the coordinating committee (contain members from the national travel health board and airport authorities).
- This research did not involve clinical or diagnostic intervention and was approved by the Travel Health Board.

Data were collected and analyzed by chi-square test. Bivariate and multivariate analyses with conditional logistic regression models were then performed on the outcome variable of visitors to the THC with significant or important input variables. Analysis was performed using the Statistical Package of Social Science (SPSS) version 16, with the level of significance set at *P* <0.05.

## Results

**Fig. 1:**
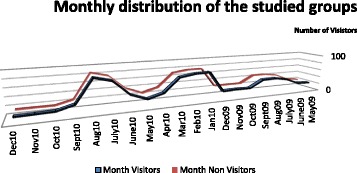
Montly distribution of the studied groups

Figure 1 presents the monthly distributions of both visitor and non-visitor groups, with two peaks in visits to the THC during July and August (summer months) and in the winter months (January, February, and March).

Table 1 shows a descriptive overview regarding some socio-demographic and travel related data among the studied groups. Male gender and active age group (30–45 years) predominate in both groups. White collar occupation are the major occupations for both groups (47.2 % for visitor group and 37.4 % for non-visitors). 59.5 % from visitors and 44.3 % from non-visitors were traveling for the leisure purpose. African countries are the major travel destination for both groups (47 % for the visitors and 34.5 for the non-visitors). Both group are planned to go to urban locations predominantly during their travel. Regarding accommodation planned, 64 % of visitors tend to stay in hotels while 48.6 % of non-visitors plan to stay with their families. Most of visitors (52.5 %) tend to stay between 1–4 weeks however 43.1 % from non-visitors tend to stay more than 4 weeks. Most of visitors and non-visitors had a history of past travels between 1–4 times. 57.3 % from Visitors got their knowledge of THC from sources other than media however 37.6 % from non-visitor had no previous knowledge of THC.

**Table 1:** Some socio-demographic and travel related data among the studies groups

Variables		Visitor	Non-Visitor
		*N* = 755	*N* = 800
Gender			
	Male	427 (56.6)	457 (57.1)
	Female	328(43.3)	343(42.9)
Nationality			
	Kuwaiti	515 (68.2)	436 (54.5)
	Non K	240 (31.8)	364 (45.5)
Age Group			
	5-30	130 (17.2)	122 (15.3)
	30-45	254 (33.7)	303 (37.9)
	45-60	185 (24.5)	201 (25.1)
	>60	186 (24.6)	174 (21.7)
Occupation			
	No work	122 (16.2)	84 (10.5)
	White collar	356 (47.2)	299 (37.4)
	Blue collar	137 (18.1)	296 (37.0)
	Domestic Worker	140 (18.5)	121 (15.1)
Purpose of Travel			
	Family	115 (15.2)	311 (38.9)
	Work	191 (25.3)	135 (16.8)
	Leisure	449 (59.5)	354 (44.3)
Travel Destination			
	Asia	196 (25)	208 (25)
	Africa	355 (47)	276 (34.5)
	S. America	76 (10)	50 (6.25)
	Others	128 (17)	266 (33.25)
Locations Visiting			
	Forest	154 (20.4)	60 (07.5)
	Rural	248 (32.8)	328 (41.0)
	Urban	353 (46.8)	412 (51.5)
Accommodations			
	Family	118 (15.6)	389 (48.6)
	Camp	154 (20.4)	144 (18.0)
	Hotel	483 (64.0)	267 (33.4)
Duration			
	<1 week	202 (26.8)	172 (21.5)
	1-4 weeks	397 (52.5)	283 (35.4)
	4-12 weeks	156 (20.7)	345 (43.1)
History of Past Travel			
	1-4 visits	533 (70.6)	612 (76.5)
	>4 visits	222 (29.4)	188 (23.5)
Knowledge of the THC			
	No	110 (14.6)	301 (37.6)
	Yes from the media	212 (28.1)	216 (27.0)
	Yes from other sources	433 (57.3)	283 (35.4)

Table 2 presents a bivariate analysis demonstrating that Kuwaiti nationality (68.2 %) significantly affects THC visits (*P* < 0.001) (OR = 1.79). Participants traveling for work (25.3 %) or leisure (59.5 %) tended to receive pre-travel consultation at the THC (*P* < 0.001) (OR = 3.83 and 3.43, respectively). Traveling to Africa and/or South America prompted travelers to visit the THC for pre-travel consultation (*P* < 0.05) (OR = 1.37 and 1.61, respectively). Travelers staying in hotels (64.0 %) and/or camps (20.4 %) were more likely to visit the THC for pre-travel consultation (*P* < 0.001). Previous knowledge of the services provided by the THC, either from the media or other sources, affected travelers’ tendencies to visit the THC for pre-travel consultation more than those with no knowledge of the THC (*P* < 0.001) (OR = 2.69 and 4.19, respectively). Blue collar employees, those with a past history of visiting more than four times, and those visiting rural or urban areas and staying between 4–12 weeks were least likely to visit the THC (*P* < 0.001). Non-significant factors included gender and age (*p* > 0.05).

**Table 2:** Bivariate analysis of the factors influencing THC visits

Variables		Visitor	Non-Visitor	P value	OR (±95 % CI)
		*N* = 755	*N* = 800		
Gender					
	Male^a^	427 (56.6)	457 (57.1)	0.8	1.02(0.84,1.25)
	Female	328(43.3)	343(42.9)		
Nationality					
	Kuwaiti^a^	515 (68.2)	436 (54.5)	<0.001	1.79 (1.46, 2.20)
	Non K	240 (31.8)	364 (45.5)		
Age Group					
	5-30^a^	130 (17.2)	122 (15.3)		1
	30-45	254 (33.7)	303 (37.9)	0.11	0.79(0.58,1.06)
	45-60	185 (24.5)	201 (25.1)	0.36	0.86(0.63,1.19)
	>60	186 (24.6)	174 (21.7)	0.98	1.00(0.73,1.38)
Occupation					
	No work^a^	122 (16.2)	84 (10.5)		1
	White collar	356 (47.2)	299 (37.4)	0.22	0.82(0.60,1.13)
	Blue collar	137 (18.1)	296 (37.0)	<0.001	0.32(0.23,0.45)
	Domestic Worker	140 (18.5)	121 (15.1)	0.22	0.80(0.55,1.15)
Purpose of Travel					
	Family^a^	115 (15.2)	311 (38.9)		1
	Work	191 (25.3)	135 (16.8)	<0.001	3.83(2.81,5.20)
	Leisure	449 (59.5)	354 (44.3)	<0.001	3.43(2.66,4.43)
Travel Destination					
	Asia^a^	196 (25)	208 (25)		1
	Africa	355 (47)	276 (34.5)	<0.05	1.37(1.06,1.75)
	S. America	76 (10)	50 (6.25)	<0.05	1.61(1.07,2.42)
	Others	128 (17)	266 (33.25)	<0.001	0.51(0.38,0.68)
Locations Visiting					
	Forest^a^	154 (20.4)	60 (07.5)		1
	Rural	248 (32.8)	328 (41.0)	<0.001	0.29(0.21,0.41)
	Urban	353 (46.8)	412 (51.5)	<0.001	0.33(0.24,0.46)
Accommodations					
	Family^a^	118 (15.6)	389 (48.6)		1
	Camp	154 (20.4)	144 (18.0)	<0.001	3.52(2.59,4.79)
	Hotel	483 (64.0)	267 (33.4)	<0.001	5.96(4.62,7.69)
Duration					
	<1 week^a^	202 (26.8)	172 (21.5)		1
	1-4 weeks	397 (52.5)	283 (35.4)	0.17	1.19(0.93,1.54)
	4-12 weeks	156 (20.7)	345 (43.1)	<0.001	0.39(0.29,0.51)
History of Past Travel					
	1-4 visits^a^	533 (70.6)	612 (76.5)	0.008	0.74 (0.59,0.92)
	>4 visits	222 (29.4)	188 (23.5)		
Knowledge of the THC					
	No^a^	110 (14.6)	301 (37.6)		1
	Yes from the media	212 (28.1)	216 (27.0)	<0.001	2.69(2.01,3.59)
	Yes from other sources	433 (57.3)	283 (35.4)	<0.001	4.19(3.21,5.46)

^a^Reference value for the comparison

As identified by multivariate analysis (Table 3), significant factors from bivariate analysis were used to establish predictors for receiving pre-travel consultation from the THC. Nationality, followed by the purpose of travel, the length of stay, and the choice of travel destination are predictors for visiting the THC (OR = 8.7, 2.2, 1.4, and 1.33, respectively).

**Table 3:** Multivariate analysis of the factors influencing THC visits.

Variables	OR	95 % CI	P
Nationality	8.7	(4.82, 13.68)	<0.001
Blue Collar Occupation	1.0	(0.93, 1.23)	>0.05
Purpose of Travel	2.2	(1.25, 4.00)	0.007
Travel Destination	1.33	(1.18, 1.42)	<0.001
Locations Visiting	0.7	(0.56, 0.79)	<0.001
Accommodation	1.4	(0.88, 1.91)	>0.05
Duration (4–12 weeks)	1.4	(1.22,1.66)	<0.001
History of Previous Travel	0.1	(0.08, 0.24)	<0.001
Knowledge of the THC	0.6	(0.39, 0.93)	0.02

Table 4 presents the preventive measures provided to visitors to the THC. Travelers to Africa and South America received yellow fever vaccination. Meningococcal vaccination was administered to a range of travelers (60.3 %- 98.4 %). One-third to two-thirds of the travelers to Asia, Africa, and S. America received typhoid and tetanus toxoid immunizations. The influenza vaccine was given to 10 % to 47.7 % of travelers to all destinations, except Africa. For malaria chemoprophylaxis, mefloquine was administered to 50.7 % of travelers and doxycycline (vibramycine or doxydar) was administered to 26.6 % of travelers to Asia, Africa, and S. America. General health education messages were given to 95.7 % of visitors, including area-specific health education messages (76.4 %).

**Table 4:** Preventive measures provided to THC visitors

Preventive measures	Total		Asia		Africa		S. America		Others	
	No	%	No	%	No	%	No	%	No	%
	755	100	196	100	355	100	76	100	128	100
• Yellow Fever	431	57.1	-	-	355	100	76	100	-	-
• ACWY meningococcal vaccine	685	90.7	193	98.4	348	98.0	63	82.9	81	63.3
• Typhoid	246	32.6	103	52.6	112	31.5	31	40.8	-	-
• Tetanus	311	41.2	73	37.2	213	60.0	25	32.9	-	-
• Influenza	122	16.2	40	20.4	-	-	21	27.6	61	47.7
• Malaria Drugs										
- Mefloquine	383	50.7	139	70.9	201	56.6	43	56.6	-	-
- Vibramycin	201	26.6	14	07.1	154	43.4	33	43.4	-	-
• Health Education										
- General HE message	723	95.7	193	98.4	355	100	76	100	99	77.3
- Specific HE message	577	76.4	160	81.6	312	87.9	43	56.6	62	48.4

## Discussion

There are two peaks during the year when more travelers visit the THC: July-August and Jan-March, due to school and university vacations during the summer, midterm, and spring periods [4].

This study found that visitors receiving pre-travel consultations at the THC were 56 % male, which differs from results reported in Quebec [8], Singapore [9], and German [9] studies. These differences might be the result of differences in gender ratios in these countries [3, 17–19].

Nationality significantly affected the likelihood of visiting the THC to receive pre-travel consultation (*P* < 0.001), which may reflect the higher per capita income of Kuwaitis and the fact that most health services are provided free of charge to Kuwaitis.

Travelers’ ages did not affect their tendency to visit the THC for pre-travel consultation (*p* > 0.05), although most travelers were from physically and financially active age groups (30–45 years and 45–60 years) [20, 21]. This finding was in agreement with the results of studies from Quebec [8], Singapore [9], and Germany [19].

Our study and a Singapore study [9] found that white collar employees visited the THC more often than individuals with other occupations, which may be attributed to their higher educational levels and higher per capita income, as reported in a Quebec study [8]. However, these differences were not significant.

Our study demonstrates that those traveling for leisure and work visited the THC more than others (*P* < 0.001), which is comparable to results reported by Vernon J. Lee and colleagues in 2006 [9] in Singapore and Susanna Flec and colleagues in 2005 [19] in Germany. This relationship might be due to an association between travel purpose and a greater likelihood of exposure to travel health hazards.

Travel destinations in Africa and South America significantly affected travelers’ tendency to visit the THC (*P* < 0.05), which is consistent with the results of other studies [5, 9, 19]. This finding might be attributed to the fact that visitors perceived the destination-specific risks of Africa and South America as higher, and so sought pre-travel health consultation at the THC; traveling to Asia, Europe, or North America was not perceived as high risk [5, 22–25].

Travelers staying in hotels visited the THC more often (*p* < 0.001), similar to results found in the Singapore study [9] in 2006, which could be attributed to the higher social class of these travelers and/or the request of travel agencies for International Travel Certificates and travel advice [16, 26].

Travelers with stays longer than 4 weeks were more likely to be frequent travelers and were less likely to visit the THC (*p* < 0.001), as was found by other studies [5–7, 9, 19]. Frequent travelers may become reluctant to seek preventive measures due to their earlier travel experiences or may have previously received travel health advice and immunizations.

Previous knowledge of the services provided by the THC significantly affected its utilization, as was also found by Vernon J Lee in Singapore [9].

Multivariate analysis identified predictors of THC attendance, including nationality, purpose of travel, accommodations, length of stay, and travel destination. Similar results were reported by the study performed in Singapore [9].

The provision of preventive services could be attributed to travelers’ perceptions of destination-specific risks and the WHO recommendations on the IHR (2005) that place greater priority on diseases such as meningococcal meningitis, yellow fever, and malaria prophylaxis for specific destinations [16, 26]. These results differ from those of another study performed in Singapore in 2006 [9], in which travelers most commonly received typhoid vaccines, hepatitis A vaccines, and D-T toxoid. This difference might be due to difference in travel destinations as in the Singapore study, the travelers traveled to Asian countries principally however in our study the traveled to African countries principally.

## Conclusion and Recommendations

The present study examined factors affecting visits to the THC and provided a clear perspective of travelers’ profiles and the services provided by the THC in Kuwait. Nationality, purpose of travel, length of stay, and travel destination are predictors for receiving a pre-travel consultation from the THC. More studies of the travel habits of Gulf Countries populations are required, especially with the increasing affluence and diverse travel habits of the region.

Health education programs must be thoroughly and extensively designed and directed to the targeted population by various educational means (mass media, audiovisuals, newspapers, magazines, posters, etc.).

### Strengths and Limitations

As the only THC providing pre-travel health consultation in Kuwait, we were able to interview all visitors to the THC, which helped us to avoid any selection bias.

All required resources and staff working in the THC were available, including physicians, health inspectors, and nurses).

We would like to highlight some limitations of this study to offer a reference for further research. This study was conducted when the (H1N1) flu pandemic was first detected in Mexico and America, and its impact greatly affected travel and was reflected in the number of travelers visiting the THC [27].

The THC provides its services during the morning working hours from 7:30 AM to 1:30 PM, which may limit the number of travelers visiting the THC, who may work during these hours.

### Ethics approval

This research did not involve clinical or diagnostic intervention and was approved by the Travel Health Board.

## Additional file

Additional file 1:
**Modified Pre-departure Health Questionnaire.**

## Abbreviations

THC: Travel Health Clinic
IHR: International Health Regulations
SPSS: Statistical Packages of Social Science
WHO: World Health Organization
D.T.: Diphtheria and Tetanus Toxoid
