# Supplementary material for: Travel risk behaviors as a determinants of receiving pre-travel health consultation and prevention
Source: Trop Dis Travel Med Vaccines. 2015 Jul 29;1:3. doi: 10.1186/s40794-015-0003-8 (PMC5526366; doi:10.1186/s40794-015-0003-8)
Supplement: Additional file 1: — Modified Pre-departure Health Questionnaire. [file 40794_2015_3_MOESM1_ESM.doc]

**Additional file**

# **Modified Pre-departure Health Questionnaire**

## Personal Data: No. (……)

**Name (Optional):**

**Gender: Nationality:**

**DOB: Occupation:**

**Residence: Education:**

## Travel Related Data:

**Travel Destination ……….**

**Purpose of Travel ………..**

**Location visiting ……….**

**Accommodation ………**

**Duration of Stay ……….**

## Past Travel History Data:

**History of Past Travel (Yes No)**

**If Yes,**

**How many travel visits …….**

**Knowledge about THC and services (Yes No)**

**If Yes,**

**From Where you got this knowledge ……**
